# Didymin mitigates neuroinflammation and preserves blood–brain barrier integrity after subarachnoid hemorrhage

**DOI:** 10.3389/fneur.2026.1857779

**Published:** 2026-06-19

**Authors:** Yingqiang Zhong, Hong Yu, Yang Wang, Jianbing Bo

**Affiliations:** Department of Neurosurgery, 3201 Hospital of Xi’an Jiaotong University Health Science Center, Hanzhong, Shaanxi, China

**Keywords:** blood–brain barrier, didymin, matrix metalloproteinase-9, neuroinflammation, subarachnoid hemorrhage

## Abstract

**Objective:**

Subarachnoid hemorrhage (SAH) is a highly lethal and disabling type of stroke. The main causes of poor prognosis are neuroinflammation, blood–brain barrier (BBB) disruption and brain edema following hemorrhage. Didymin has shown neuroprotective effects in intracerebral hemorrhage; however, its regulatory role in SAH remains unclear.

**Methods:**

The rat SAH model was established using the internal carotid artery puncture method, while an *in vitro* model was developed by stimulating human brain microvascular endothelial cells (HBMECs) with hemoglobin (Hb). Following didymin treatment, neurological functional outcomes were assessed using the modified Garcia score and the balance beam test. Nissl staining was performed to evaluate neuronal pathological changes. Immunofluorescence staining was employed to assess microglial activation and BBB integrity. Brain water content was measured to evaluate the severity of cerebral edema. Western blot analysis was utilized to detect the expression of matrix metalloproteinase 9 (MMP9), apoptosis-related proteins (Bcl-XL, Bcl-2, Bax), pro-inflammatory cytokines (IL-1β, IL-6, TNF-*α*), and tight junction proteins (ZO-1, Occludin).

**Results:**

Didymin treatment significantly improved neurological function scores in SAH rats by alleviating neuronal damage and apoptosis. On one hand, didymin reduced post-SAH neuroinflammation by inhibiting excessive microglial activation and the expression of pro-inflammatory cytokines. On the other hand, didymin preserved BBB integrity and alleviated brain edema by downregulating MMP9 expression. In Hb-induced cell model, didymin suppressed MMP9 expression and promoted the expression of tight junction proteins.

**Conclusion:**

In this study, we demonstrated that didymin mitigates neuronal damage and apoptosis following SAH, effectively suppresses neuroinflammation, maintains the integrity of the BBB, and attenuates brain edema. These findings suggest that didymin holds promise as a potential therapeutic candidate for the treatment of SAH.

## 1 Introduction

Subarachnoid hemorrhage (SAH), predominantly caused by the rupture of intracranial aneurysms, is a devastating cerebrovascular event that accounts for approximately 5 to 10% of all strokes worldwide (1). Strikingly, SAH predominantly affects individuals aged 40 to 60, leading to a disproportionately high loss of productive life years and a massive socioeconomic burden (2). Despite contemporary advancements in neurosurgical and endovascular interventions, the clinical prognosis remains suboptimal, with mortality rates approaching 40% and over one-third of survivors suffering from permanent neurological deficits (3, 4). A major reason for poor outcome is the development of early brain injury and subsequent secondary injury cascades, necessitating the development of novel therapeutic strategies targeting the multi-layered mechanisms of secondary brain injury.

Among the secondary injury mechanisms, neuroinflammation and BBB dysfunction are especially important and closely interconnected (5, 6). Following SAH, activated microglia and infiltrating immune cells release large amounts of pro-inflammatory mediators, which amplify tissue injury and exacerbate neuronal damage. In parallel, BBB integrity is compromised through degradation of tight junction proteins and extracellular matrix components, leading to increased vascular permeability, vasogenic edema, and worsened neurological deficits (7–10). Matrix metalloproteinases, particularly MMP9, have been implicated in BBB breakdown after SAH by promoting tight junction disruption and endothelial injury (11, 12). The subsequent breach in BBB integrity facilitates the infiltration of peripheral immune cells and blood-derived neurotoxins into the brain parenchyma, culminating in vasogenic edema and increased intracranial pressure (13). This interaction between neuroinflammation and BBB breakdown exacerbates neuronal apoptosis and neurological impairment, identifying the stabilization of the BBB and the suppression of the inflammatory cascade as pivotal therapeutic objectives.

Didymin is a naturally occurring flavonoid glycoside that has attracted increasing attention for its anti-inflammatory, antioxidant, and neuroprotective properties (14). Previous studies have reported that didymin exerts beneficial effects in several central nervous system injury models, including cerebral ischemia–reperfusion injury and intracerebral hemorrhage, where it reduces inflammatory responses, attenuates neuronal damage, and improves functional recovery (15, 16). However, whether didymin is protective in SAH remains unclear. In the present study, we therefore investigated the effects of didymin on neurological outcomes, neuroinflammation, and BBB integrity after SAH, and further explored whether its protective actions are associated with inhibition of MMP9-mediated BBB disruption. We aimed to provide experimental evidence supporting didymin as a potential therapeutic strategy for SAH.

## 2 Materials and methods

### 2.1 SAH model

Adult male Sprague–Dawley rats (250–300 g) were fasted for 12 h before surgery, with free access to water. Anesthesia was induced via intraperitoneal sodium pentobarbital (20 mg/kg). Sodium pentobarbital was prepared as a 3% solution (30 mg/mL) in sterile saline. In the supine position, a midline cervical incision (~2 cm) was made to expose the right common carotid artery (CCA), internal carotid artery (ICA), and external carotid artery (ECA). The proximal CCA and distal ECA were clamped, and a lubricated nylon filament was inserted into a small incision in the proximal ICA, advanced to the skull base, and used to puncture the ICA bifurcation, inducing SAH. After hemostasis and suturing, the rats recovered in a temperature-controlled chamber. After completion of the experimental procedures, all animals were euthanized via intraperitoneal injection of an overdose of sodium pentobarbital (≥150 mg/kg). The absence of heartbeat and respiratory arrest was confirmed prior to tissue collection. This method complies with the AVMA Guidelines for the Euthanasia of Animals (2020 edition). All experimental protocols were reviewed and approved by the Xi’an Jiaotong University (K202415-1) in accordance with the ARRIVE guidelines. This study was conducted in strict compliance with ethical standards.

### 2.2 SAH grade and neurological score

SAH severity was assessed using a well-established scoring system. Briefly, the ventral surface of the brain was divided into six regions based on anatomical landmarks: (1) the olfactory bulb and anterior cranial base, (2) the circle of Willis and middle cerebral artery territory, (3) the optic chiasm and hypothalamic region, (4) the ventral midbrain, (5) the pons, and (6) the medulla and ventral cerebellum. Each region was scored from 0 to 3 according to the degree of subarachnoid blood clotting: 0, no hemorrhage; 1, minimal scattered bleeding; 2, moderate hemorrhage partially covering the region; 3, extensive hemorrhage filling the entire region. The total SAH score was calculated as the sum of scores from all six regions, with higher scores indicating greater bleeding severity. Rats with a total SAH score of less than 7 were excluded.

The modified Neurological Severity Score (mNSS) was assessed at 24 h after SAH modeling. All experimental SD rats were randomly assigned to corresponding groups. The total score ranges from 0 to 18, with higher scores representing more serious neurological damage. All behavioral assessments were conducted in triplicate, and average values were adopted for statistical analysis.

Neurological performance was evaluated 24 h post-SAH using the modified Garcia score (17) and the beam balance test. The modified Garcia score assesses six parameters: spontaneous activity, independent movement of all four limbs, forepaw extension, whisker proprioception, body proprioception, and climbing ability. Each category is scored from 0 to 3, with the total score being the sum of all components. The beam balance test was conducted to measure the rats’ balance coordination. Specifically, rats were placed on a narrow wooden beam and observed for 1 min; scores ranged from 0 to 4 based on the distance traversed and the frequency of falls or slips.

### 2.3 Drug administration

Didymin (14259-47-3, MCE) was dissolved in 1% dimethyl sulfoxide (DMSO) to prepare a stock solution (1.5 mg/mL) and administered via intraperitoneal (i.p.) injection. Based on preliminary dose-ranging experiments, 1.5 mg/kg was selected as the optimal intervention dose for all subsequent efficacy assessments. The injection volume was calculated according to each rat’s body weight (approximately 200 μL for a 200 g rat). The vehicle group received an equivalent volume of 1% DMSO. All treatments were performed 1 h after SAH induction, which was determined to be the optimal time point for drug administration. Cells were pre-treated with 20 μM Hb for 24 h to establish the injury model. Subsequently, didymin (20 μM), the NF-κB activator PMA (MCE, HY-18739, 20 ng/mL), and the RKIP inhibitor Locostatin (MCE, HY-W013411A, 50 μM) were co-administered for an additional 24 h.

### 2.4 Brain water content

Rats were sacrificed 24 h after surgery. The brain tissue from the injured hemisphere was collected, and its wet weight was measured and recorded. The tissue was then dried at 100 °C for 24 h, and the dry weight was measured and recorded. Brain water content was calculated using the following formula: [(wet weight − dry weight)/wet weight] × 100%.

### 2.5 Nissl staining

Fourteen days after SAH modeling, rats were sacrificed and transcardially perfused with cold PBS by 4% paraformaldehyde to achieve tissue fixation. The brains were subsequently immersed in PFA at 4 °C for 24 h. After fixation, the brain tissues were embedded in paraffin and sectioned into 5-μm thick slices for downstream analyses. On day 14 post-SAH, Nissl staining was performed using 5% cresyl violet solution, and neuronal structure and cell density were evaluated under a light microscope.

### 2.6 Immunofluorescence staining

Rats were perfused with ice-cold PBS and 4% PFA 24 h after SAH. Brain samples were fixed in PFA for 24 h, dehydrated in 30% sucrose for 3 days, and then embedded in OCT before freezing at −80 °C. Cryostat sections were prepared for double immunofluorescence. For immunofluorescence, slides were blocked with 5% BSA for 1 h and incubated overnight at 4 °C with primary antibodies: anti-CD31 (1:100, ab222783, Abcam), anti-MMP9 (1:400, 10375-2-AP, proteintech), anti-Iba1 (1:200, 17198, Cell Signaling Tech). The sections were washed with PBS and incubated with a secondary antibody for 2 h at room temperature.

### 2.7 Western blotting

Proteins were extracted with RIPA lysis solution (Beyotime, China) the protocols were performed in accordance with the manufacturer’s instructions. The protein sample loaded in an equal amount and transferred to the polyvinylidene fluoride membrane after separation by SDS-PAGE electrophoresis. The membrane was incubated overnight at 4 °C with the following primary antibodies: anti-MMP9 (1:400, 10375-2-AP, proteintech), anti-Bcl-XL (1:2000, 10783-1-AP, proteintech), anti-Bcl-2 (1:500, WL01556, Wanleibio), anti-Bax (1:2000, ab32503, Abcam), anti-IL-6 (1:1000, ab259341, Abcam), anti-IL-1*β* (1:1000, ab254360, Abcam), anti-TNF-*α* (1:1000, ab255275, Abcam), anti-β-actin (1:2000, ab8226, Abcam), anti-ZO-1 (1:5000, 21773-1-AP, proteintech), anti-Occludin (1:5000, 27260-1-AP, proteintech). The membranes were incubated with the corresponding secondary antibodies and, after being washed three times, were visualized using an enhanced chemiluminescence reagent kit. The resulting images were processed and analyzed using ImageJ software.

### 2.8 Cell viability

Cell Counting Kit-8 (C0037, Beyotime) was employed to assess cytotoxicity. For the CCK-8 assay, cells were treated with didymin at different concentrations of 2, 5, 10, 20 and 50 μM for 24 h, and the experimental procedure was carried out in strict accordance with the manufacturer’s instructions.

### 2.9 Real-time quantitative PCR

Total RNA was extracted using TRIzol reagent (279510, Thermo Fisher Scientific). After quantification with a NanoDrop 2000C spectrophotometer (Thermo Fisher Scientific), reverse transcription was performed using the Transcriptor First Strand cDNA Synthesis Kit (Roche). qPCR was carried out with SYBR Green Premix (Roche) as the fluorescent dye. The relative gene expression level was normalized with the *β*-actin using the comparative cycle threshold (Ct) method (2^−ΔΔCt^). Primer sequences used for this study: MMP9-F: CCTGGGCAGATTCCAAACCT, MMP9-R: GCAAGTCTTCCGAGTAGTTTTGGAT; TNF-*α*-F: CCTCTCTCTAATCAGCCCTCTG, TNF-α-R: GAGGACCTGGGAGTAGATGAG; IL-1β-F: CGAATCTCCGACCACCACTA, IL-1β-R: AGGGAAAGAAGGTGCTCAGG; ACTB-F: CATGTACGTTGCTATCCAGGC, ACTB-R: CTCCTAATGTCACGCACGAT.

### 2.10 Statistical analysis

The sample size was calculated *a priori* using SAS 9.1 software (SAS Institute Inc.), with the modified Garcia score at 24 h after SAH serving as the primary endpoint for the power analysis. Based on previous studies of didymin treatment in stroke models and behavioral assessments (16), an expected between-group difference of 3 and a standard deviation of 1.5 were used for calculation. Using a two-sided *α* of 0.05 and a power of 0.80 with an allocation ratio of 1:1 among groups, the minimum required sample size was approximately 4 rats per group. To ensure robustness and account for potential biological variability, the sample size was determined with reference to a previous study (18). Considering an estimated mortality and exclusion rate of 20–25% in the SAH model, we ultimately used *n* = 6 per group for behavioral assessments and *n* = 5 per group for biochemical analyses (The specific sample sizes are detailed in Supplementary Table S1).

Shapiro–Wilk Test was used to determine normality prior to parametric tests. Levene’s test was used for homogeneity of variance. For continuous variables with a normal distribution, data are expressed as mean ± standard deviation (mean ± SD). One-way analysis of variance (ANOVA) followed by Tukey’s *post hoc* test was used to compare data from three or more groups with one variable. For ordinal or semi-quantitative data, including SAH grade, mNSS, modified Garcia scores, and beam balance scores, data are presented as median [interquartile range (IQR)]. Statistical comparisons among groups were performed using the non-parametric Kruskal–Wallis test followed by Dunn’s *post hoc* test. *p* < 0.05 was considered statistically significant. All statistical analyses were conducted using GraphPad Prism software.

## 3 Results

### 3.1 SAH model mortality and grade score

**Figure 1:**
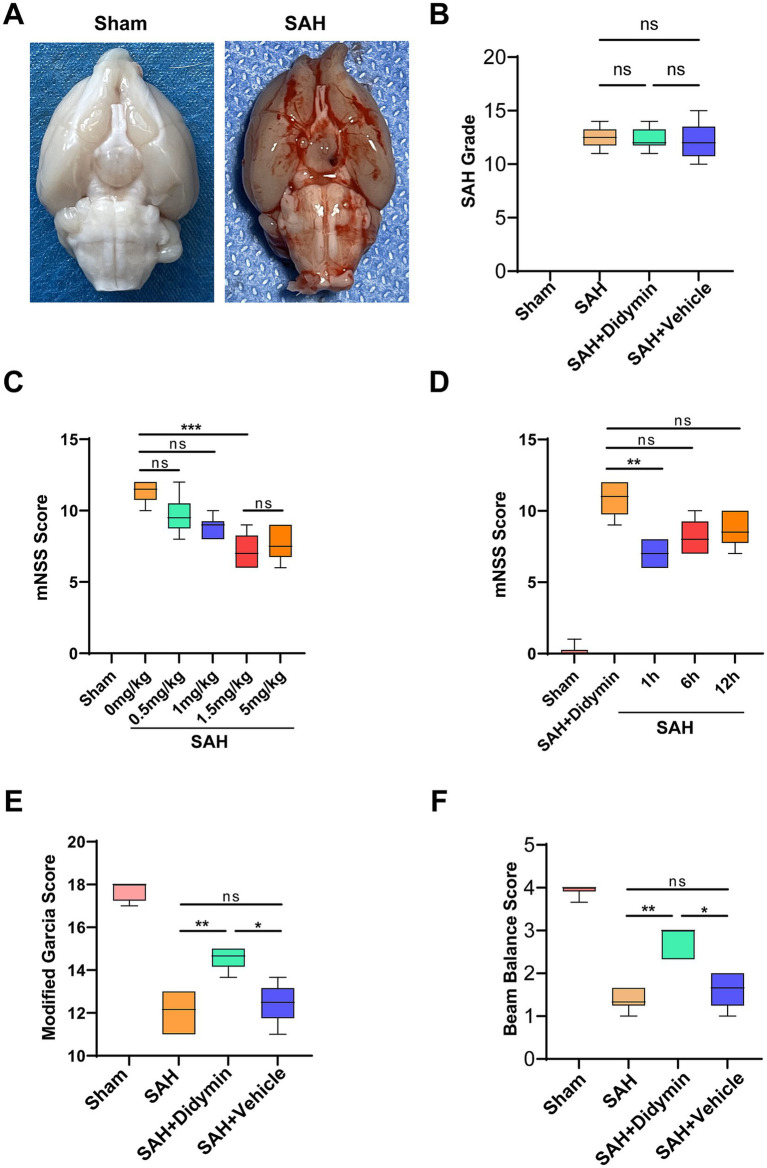
Didymin improves neurological outcomes after SAH in rats. **(A)** Representative images of the rat SAH model. **(B)** SAH grading scores among the experimental groups. **(C)** mNSS scores of rats 24 h after intervention with indicated doses of didymin. **(D)** mNSS scores of rats 24 h after didymin treatment at indicated time points. **(E)** Modified Garcia scores at 24 h after SAH. **(F)** Beam balance scores at 24 h after SAH. Data are presented as median [IQR]. *n* = 6 rats per group. Statistical comparisons among groups were performed using the non-parametric Kruskal–Wallis test followed by Dunn’s *post hoc* test. *p < 0.05; **p < 0.01; ***p < 0.001; ns, not significant.

The rat SAH model was successfully established using the internal carotid artery puncture method (Figure 1A). To ensure uniformity in hemorrhage severity following SAH and to minimize potential biases in the experimental results caused by variations in bleeding severity, post-mortem assessment of SAH bleeding grades was conducted. No significant differences were observed in the SAH grade scores among the experimental groups (Figure 1B).

### 3.2 Didymin improves neurological scores in rat after SAH

To determine the optimal therapeutic dose of didymin, we established different concentration gradients (Figure 1C) and intervention time points (Figure 1D), and evaluated the mNSS scores in rats 24 h post-intervention. The results indicated that administering didymin at a dose of 1.5 mg/kg, 1 h after SAH, represented the optimal intervention time and dosage.

Neurological function in rats following SAH was evaluated using the modified Garcia score and the beam balance test. Compared with the SAH group, didymin administration significantly improved neurological performance (Figures 1E,F). These findings suggest that didymin significantly enhances short-term neurological recovery in rats following SAH.

### 3.3 Didymin alleviates neuronal injury and apoptosis in rats after SAH

**Figure 2:**
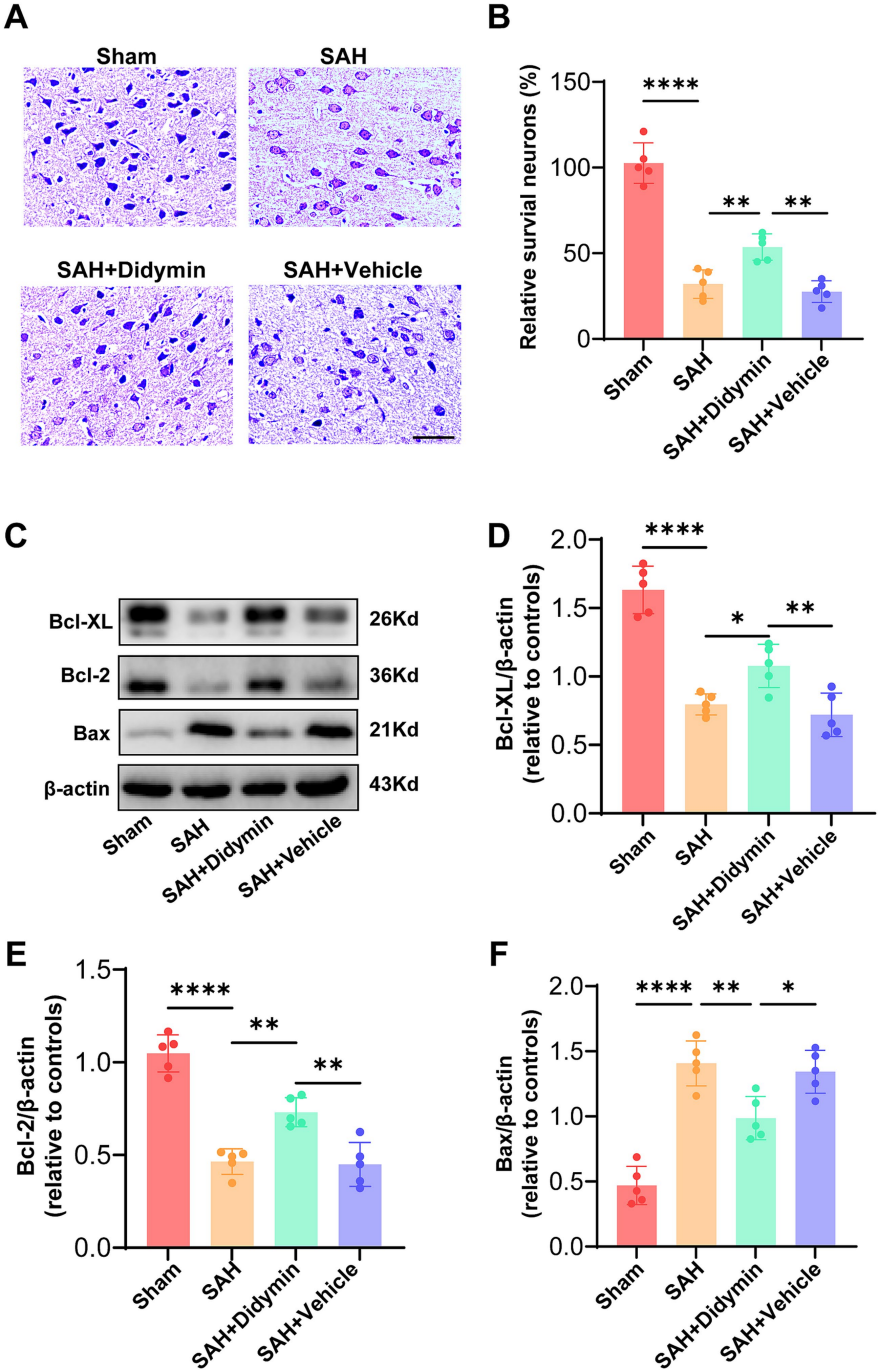
Didymin alleviates neuronal injury and apoptosis after SAH. **(A)** Representative Nissl staining images in the Sham, SAH, SAH + Didymin, and SAH + Vehicle groups. Scale bar = 50 μm. **(B)** Quantification of Nissl staining in indicated groups. **(C)** Representative Western blot images of Bcl-XL, Bcl-2, and Bax. **(D–F)** Quantification of Bcl-XL, Bcl-2, and Bax expression. Data are presented as mean ± SD. *n* = 5 rats per group. Statistical analysis was performed using one-way ANOVA followed by Tukey’s *post hoc* test. *p < 0.05; **p < 0.01; ****p < 0.0001.

To assess neuronal damage in rats following SAH, cortical neurons were evaluated using Nissl staining. Compared with the Sham group, the SAH group exhibited a significant increase in the number of damaged neurons. Conversely, didymin treatment significantly reduced the number of damaged cortical neurons (Figures 2A,B). Additionally, the expression levels of apoptosis-related proteins were analyzed across the groups. SAH markedly upregulated the expression of the pro-apoptotic protein Bax and downregulated the expression of the anti-apoptotic proteins Bcl-XL and Bcl-2. However, didymin intervention effectively reversed these changes, significantly attenuating neuronal apoptosis after SAH (Figures 2C–F). Collectively, these results indicate that didymin treatment mitigates neuronal death induced by SAH.

### 3.4 Didymin alleviates excessive microglial activation and the secretion of pro-inflammatory cytokines in rats after SAH

**Figure 3:**
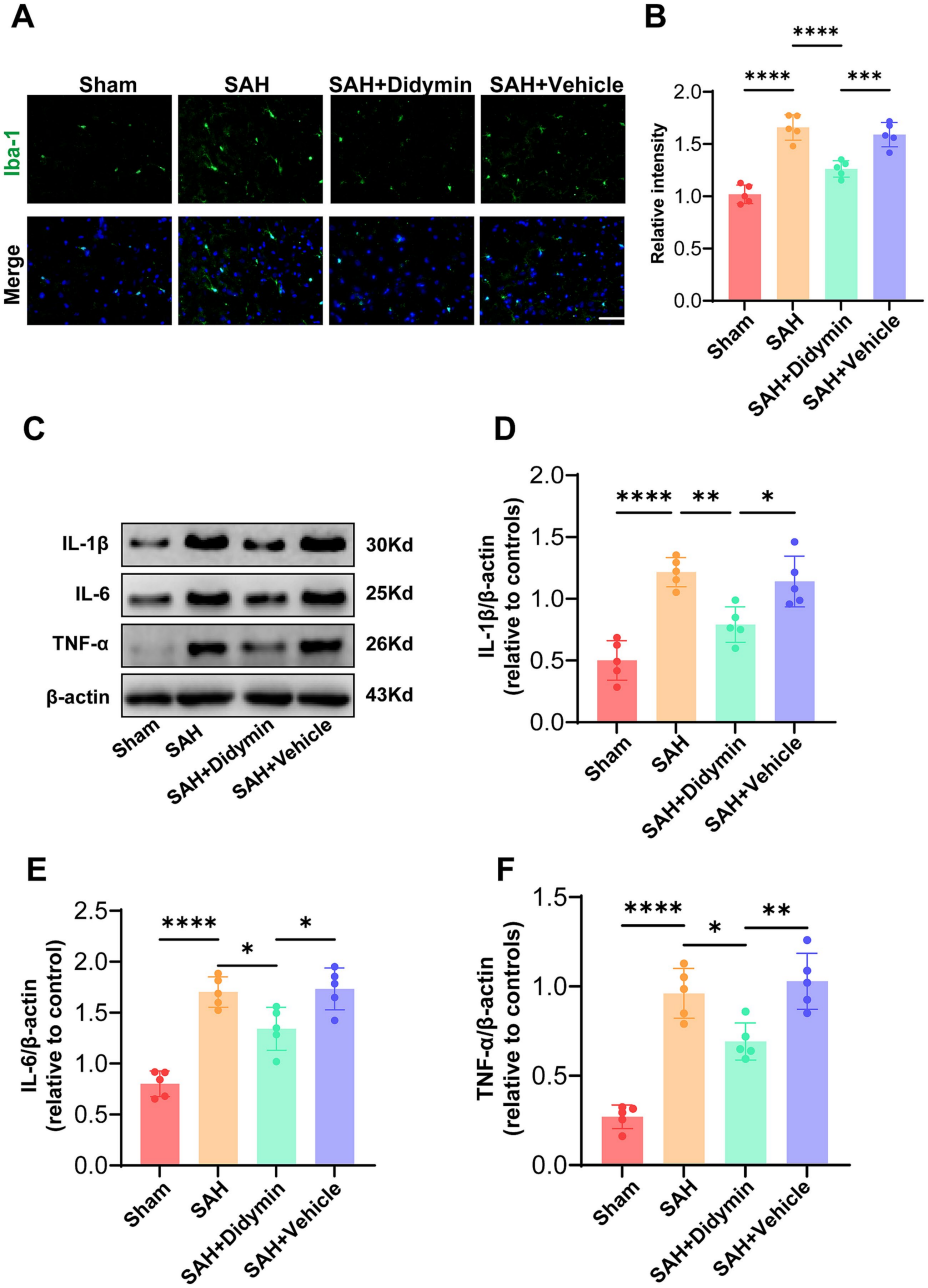
Didymin suppresses microglial activation and pro-inflammatory cytokine expression after SAH. **(A)** Representative immunofluorescence images of Iba-1-positive microglia in each group. Scale bar = 50 μm. **(B)** Quantification of Iba-1-positive microglia in indicated groups. **(C)** Representative Western blot images of IL-1β, IL-6, and TNF-α. **(D–F)** Quantification of IL-1β, IL-6, and TNF-α expression. Data are presented as mean ± SD. *n* = 5 rats per group. Statistical analysis was performed using one-way ANOVA followed by Tukey’s *post hoc* test. *p < 0.05; **p < 0.01; ***p < 0.001; ****p < 0.0001.

Given that neuroinflammation is a key determinant of neurological outcomes, we sought to evaluate whether didymin treatment could attenuate neuroinflammatory damage in rats after SAH. Immunofluorescence staining for Iba-1 demonstrated a marked increase in the number of activated microglia in the SAH group compared to the Sham group (Figures 3A,B). Notably, didymin treatment significantly decreased the number of Iba-1-positive microglia in the left temporal cortex (Figures 3A,B). To further elucidate the impact of didymin on neuroinflammation, we quantified the protein levels of pro-inflammatory cytokines IL-1β, IL-6, and TNF-*α*. Consistent with the immunofluorescence results, the expression of these cytokines was significantly elevated in the SAH group relative to the Sham group, whereas didymin treatment effectively reduced their levels (Figures 3C–F). Collectively, these findings suggest that didymin alleviates early excessive neuroinflammatory damage following SAH.

### 3.5 Didymin suppresses endothelial MMP9 expression, preserves vascular endothelial integrity, and attenuates brain edema in rats of SAH

**Figure 4:**
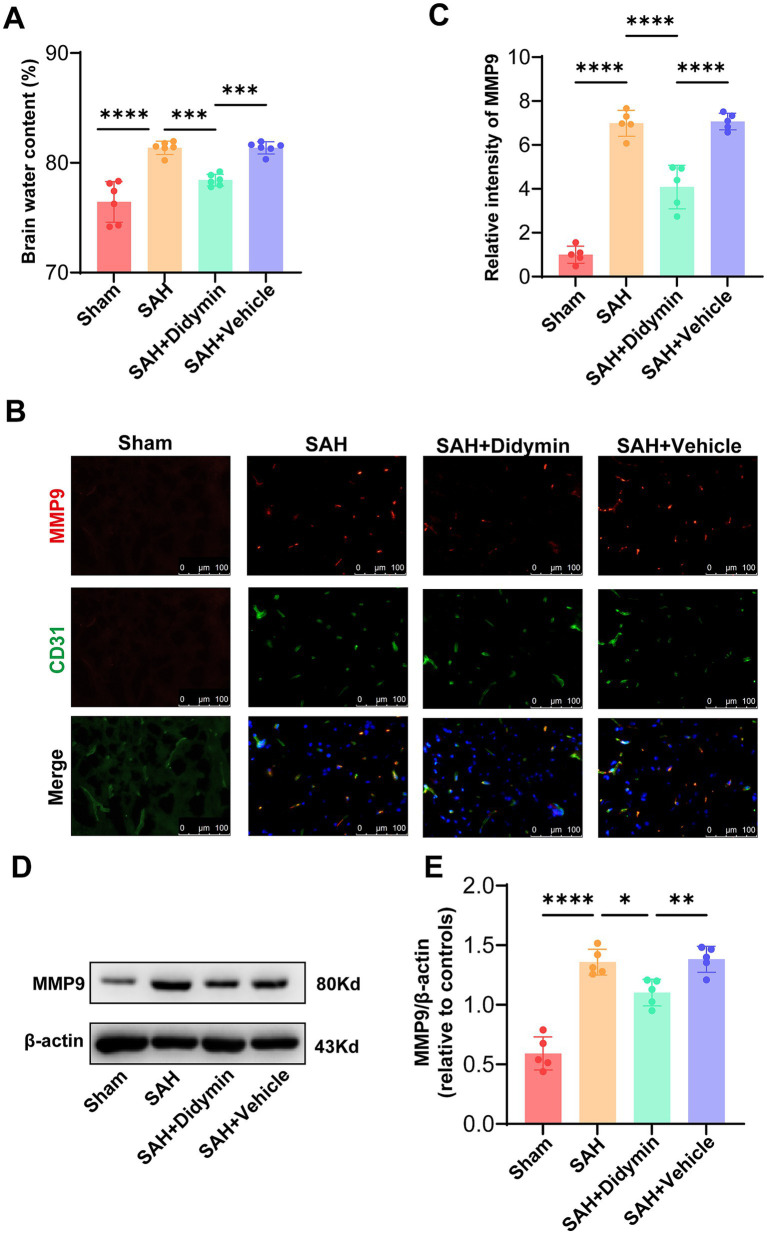
Didymin alleviates BBB disruption and brain edema after SAH by suppressing MMP9. **(A)** Brain water content in each group (*n* = 6 rats per group). **(B)** Representative confocal images showing MMP9 expression in CD31-positive endothelial cells. Scale bar = 100 μm (*n* = 5 rats per group). **(C)** Quantification of MMP9-positive endothelial cells in indicated groups. **(D)** Representative Western blot images of MMP9. **(E)** Quantification of MMP9 expression (*n* = 5 rats per group). Data are presented as mean ± SD. Statistical analysis was performed using one-way ANOVA followed by Tukey’s *post hoc* test. *p < 0.05; **p < 0.01; ***p < 0.001; ****p < 0.0001.

We assessed the effect of didymin treatment on brain edema by measuring brain water content in rats following SAH. The results demonstrated that didymin intervention significantly attenuated brain edema induced by SAH (Figure 4A). BBB disruption is a key contributor to brain edema after SAH, with MMPs playing a pivotal role by degrading the extracellular matrix and compromising BBB integrity. To explore the impact of didymin on MMP9 expression and endothelial cell integrity following SAH, immunofluorescence staining was conducted for vascular endothelial cells and MMP9. The findings revealed that MMP9 expression was markedly upregulated after SAH, whereas didymin treatment significantly reduced MMP9 expression in endothelial cells (Figures 4B,C). Additionally, CD31 staining indicated that didymin preserved vascular endothelial integrity in SAH rats (Figures 4B,C). These observations were further corroborated by Western blot analysis, which confirmed that didymin treatment significantly suppressed MMP9 expression following SAH (Figures 4D,E).

### 3.6 Didymin suppresses endothelial MMP9 expression and upregulates the expression of tight junction proteins in an *in vitro* model of SAH

**Figure 5:**
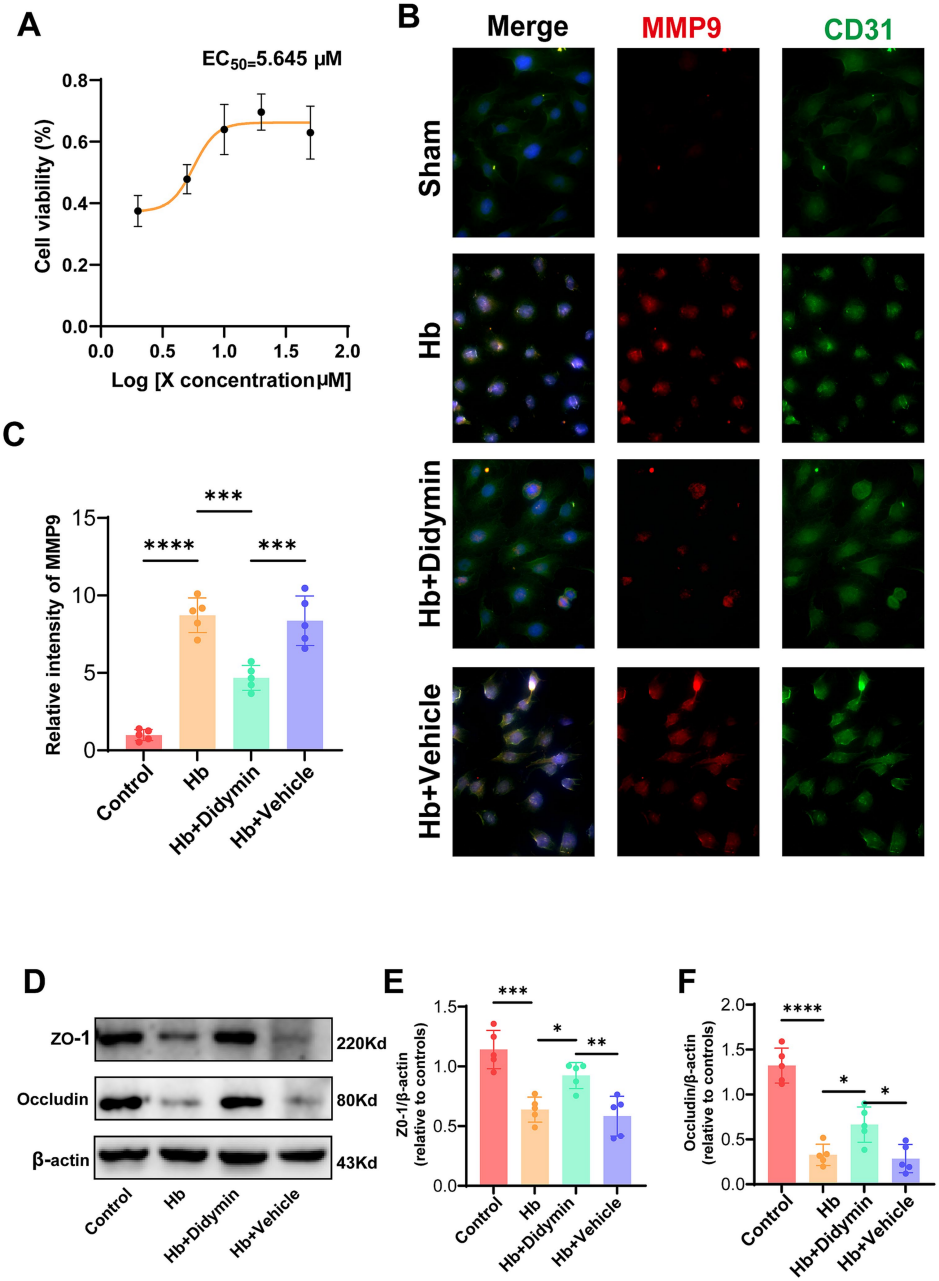
Didymin preserves tight junction protein expression in Hb-treated HBMECs. **(A)** Dose–response curve in HBMECs treated with different doses (2, 5, 10, 20, 50 μM) of didymin for 24 h after Hb induction (*n* = 6 per group). **(B)** Representative immunofluorescence images showing MMP9 expression in CD31-positive endothelial cells after Hb treatment. Scale bar = 100 μm. **(C)** Quantification of MMP9-positive endothelial cells in indicated groups (*n* = 5 per group). **(D)** Representative Western blot images of ZO-1 and Occludin. **(E–F)** Quantification of ZO-1 and Occludin expression (*n* = 5 per group). Data are presented as mean ± SD. Statistical analysis was performed using one-way ANOVA followed by Tukey’s *post hoc* test. *p < 0.05; **p < 0.01; ***p < 0.001; ****p < 0.0001.

To further validate the protective effects of didymin on the BBB following SAH, an in vitro model was established by stimulating HBMECs with Hb to mimic SAH-induced damage. To identify the optimal in vitro dose of didymin, cell viability was assessed using a concentration gradient of 2, 5, 10, 20, and 50 μM. The dose–response curve confirmed 20 μM as the optimal concentration for in vitro intervention (Figure 5A). The expression of MMP9 and tight junction proteins was assessed. Hb stimulation significantly upregulated MMP9 expression in vitro, whereas didymin treatment effectively suppressed its expression. Moreover, morphological changes in endothelial cells were observed, including cell body shrinkage (Figures 5B,C). To evaluate the impact of didymin on tight junction integrity, the expression levels of the tight junction proteins ZO-1 and Occludin were analyzed. The results demonstrated that Hb stimulation markedly inhibited the expression of ZO-1 and Occludin, while didymin intervention successfully reversed these effects (Figures 5D–F). These findings highlight the potential role of didymin in preserving endothelial integrity and tight junction structure under SAH-related conditions.

Previous studies have demonstrated that didymin exerts neuroprotective effects by inhibiting NF-κB and activating RKIP (16, 19). To investigate whether didymin alleviates BBB damage and suppresses neuroinflammation through these pathways after SAH, we separately activated NF-κB and inhibited RKIP, then detected the transcriptional levels of MMP9 and related inflammatory factors. The results revealed that compared with the didymin-treated group, the mRNA levels of MMP9 and inflammatory factors were markedly increased upon NF-κB activation or RKIP inhibition. Collectively, these observations indicate that RKIP and NF-κB may participate in the neuroprotective effects of didymin, though further validation is required to establish definitive causal relationships.

## 4 Discussion

In the present study, we found that didymin significantly improved neurological function after SAH, reduced neuronal injury and apoptosis, inhibited excessive microglial activation and pro-inflammatory cytokine secretion, and relieved cerebral edema. Mechanistically, didymin suppressed endothelial MMP9 expression and maintained tight junction integrity, indicating that its neuroprotective effects are closely associated with BBB stability.

Our findings align with and extend the growing body of evidence regarding the neuroprotective potential of didymin. Previous research has established didymin as a potent anti-inflammatory agent in various stroke models. For instance, Li et al. reported that didymin alleviates cerebral ischemia–reperfusion injury by activating the PPAR signaling pathway and reducing microglial overactivation (15). More recently, Gu et al. demonstrated that didymin suppresses microglia pyroptosis and neuroinflammation through the Asc/Caspase-1/GSDMD pathway following intracerebral hemorrhage (ICH) (16). While these studies focused on microglial polarization and cell death pathways, our study provides a novel perspective by highlighting didymin’s specific role in stabilizing the neurovascular unit. We observed that didymin not only suppresses microglial activation but also directly targets the MMP9-mediated degradation of tight junction proteins (ZO-1 and Occludin) in vascular endothelial cells. This suggests that the benefits of didymin in SAH are multifaceted, addressing both the parenchymal inflammatory response and the structural integrity of the BBB.

SAH leads to sustained elevation of intracranial pressure, which significantly exacerbates adverse neurological outcomes, primarily as a result of severe brain edema (20). The integrity of the BBB plays a pivotal role in maintaining cerebral homeostasis. Disruption of the BBB facilitates the infiltration of peripheral inflammatory mediators into the central nervous system, thereby amplifying neuroinflammatory damage and promoting aberrant neuronal apoptosis (21). A primary innovation of this study is the elucidation of the relationship between didymin and MMP9 in the context of SAH. MMP9 is a well-known executioner of BBB breakdown, and its upregulation is closely linked to the development of vasogenic edema (11, 22). Our data showed that didymin significantly downregulated MMP9 expression both in the temporal cortex of SAH rats and in Hb-stimulated HBMECs. By preserving the vascular scaffold, didymin effectively broke the vicious cycle where BBB leakage exacerbates the infiltration of peripheral inflammatory mediators, which would otherwise lead to secondary neuronal damage. This focus on the endothelial-junctional complex offers a specific mechanistic explanation for the reduction in brain water content observed in our treated groups. In addition, we further investigated the potential mechanisms. Our findings suggest that RKIP activation and NF-κB inhibition may contribute to the anti-inflammatory and anti-edema effects of didymin in the setting of SAH.

Despite the promising neuroprotective effects observed in this study, the clinical translation of didymin faces several pharmacological and safety challenges that warrant further investigation. As a natural flavonoid, didymin possesses broad biological regulatory activities *in vivo*. Beyond the specific neuroprotective mechanisms focused on in this study, it may exert non-specific regulatory effects on multiple signaling pathways. Consequently, we cannot exclude the possibility that high doses or long-term administration of didymin might induce slight off-target effects on normal neuronal metabolism, vascular endothelial function, and systemic inflammatory homeostasis in non-injured regions. Furthermore, the clinical potential of didymin is currently constrained by its pharmacokinetic limitations. Like many natural flavonoids, didymin suffers from poor water solubility, low oral bioavailability, and rapid metabolic clearance in vivo (23, 24). To overcome these shortcomings and lay a solid foundation for clinical use, future research should prioritize the optimization of drug delivery systems, such as nanoparticle encapsulation or molecular structural modification—to enhance its bioavailability and tissue targeting specificity (25).

However, several limitations in this study warrant consideration. First, we focused exclusively on the acute phase of SAH (24 h post-injury). The long-term effects of didymin on neuroregeneration, cognitive function, and delayed cerebral ischemia remain unknown. Second, although we identified the downregulation of MMP9 as a key outcome, the precise upstream molecular targets of didymin, such as specific transcription factors or cell surface receptors, were not fully identified. The use of specific inhibitors or gene-silencing techniques in future studies would be necessary to establish a direct causal link between didymin’s target and MMP9 suppression. Third, this study utilized only young adult male rats. Given that clinical SAH outcomes can be influenced by age and sex-specific hormonal factors, the generalizability of these findings to a broader population requires further validation in female and aged animal models.

## 5 Conclusion

In summary, BBB disruption following SAH is a multifactorial process driven by neuronal damage, neuroinflammation, and MMP activation. Our findings emphasize the diverse role of didymin in the pathophysiology of SAH and highlight the need for further research into its regulatory mechanisms. This study offers vital evidence to support the potential of didymin as an effective therapeutic option for SAH treatment.

## Data Availability

The raw data supporting the conclusions of this article will be made available by the authors, without undue reservation.

## Supplementary material

SUPPLEMENTARY FIGURE S1 Potential association between RKIP/NF-κB pathway and didymin-mediated neuroprotection. **(A–F)** Relative mRNA quantification of MMP9, IL-1β and TNF-α in different groups (*n* = 5 per group). Data are presented as mean ± SD. Statistical analysis was performed 464 using one-way ANOVA followed by Tukey’s *post hoc* test. ***p* < 0.01; *****p* < 0.0001.
