# Supplementary figures and images for: Didymin mitigates neuroinflammation and preserves blood–brain barrier integrity after subarachnoid hemorrhage
Source: Front Neurol. 2026 Jun 19;17:1857779. doi: 10.3389/fneur.2026.1857779 (PMC13332488; doi:10.3389/fneur.2026.1857779)

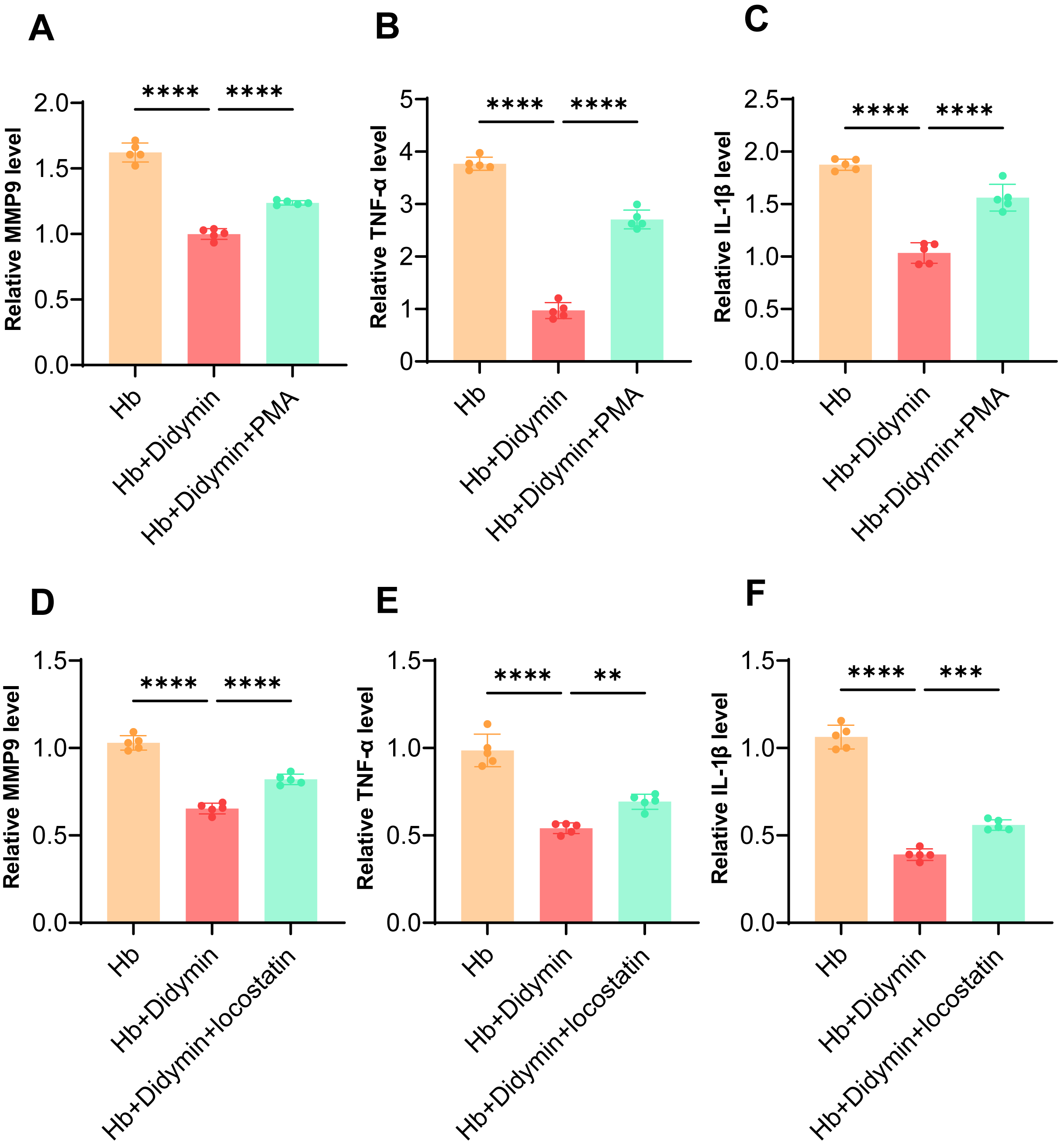

Supplement: SUPPLEMENTARY FIGURE S1 — Potential association between RKIP/NF-κB pathway and didymin-mediated neuroprotection. (A–F) Relative mRNA quantification of MMP9, IL-1β and TNF-α in different groups (n = 5 per group). Data are presented as mean ± SD. Statistical analysis was performed 464 using one-way ANOVA followed by Tukey’s post hoc test. **p < 0.01; ****p < 0.0001. [file Image_1.TIF]
