# Supplementary material for: Didymin mitigates neuroinflammation and preserves blood–brain barrier integrity after subarachnoid hemorrhage
Source: Front Neurol. 2026 Jun 19;17:1857779. doi: 10.3389/fneur.2026.1857779 (PMC13332488; doi:10.3389/fneur.2026.1857779)
Supplement: Supplementary file 2 [file Table_1.DOCX]

**Supplementary Table 1**

| **Groups** | **Mortality** | **Exclude** |
| --- | --- | --- |
| **SAH Grade** |  |  |
| Sham  SAH  SAH+Didymin  SAH+Vehicle | 0% (0/6)  11.11% (1/9)  12.5% (1/8)  14.29% (1/7) | 0  2  1  0 |
| **Evaluation of optimal intervention dose** |  |  |
| Sham  SAH  SAH+Didymin (0.5 mg/kg)  SAH+Didymin (1 mg/kg)  SAH+Didymin (1.5 mg/kg)  SAH+Didymin (5 mg/kg) | 0% (0/6)  22.22% (2/9)  20% (2/10)  14.29% (1/7)  12.5% (1/8)  12.5% (1/8) | 0  1  2  0  1  1 |
| **Evaluation of Optimal intervention time points** |  |  |
| Sham  SAH  SAH+Didymin (1 h)  SAH+Didymin (6 h)  SAH+Didymin (12 h) | 0% (0/6)  22.22% (2/9)  11.11% (1/9)  20% (2/10)  12.5% (1/8) | 0  1  2  2  1 |
| **Evaluation of neurological function, neuroinflammation (Iba-1 staining) and blood-brain barrier integrity (MMP9 staining)** |  |  |
| Sham  SAH  SAH+Didymin  SAH+Vehicle | 0% (0/6)  20% (2/10)  11.11% (1/9)  30% (3/10) | 0  2  2  1 |
| **Evaluation of neuronal apoptosis (WB, BCL-XL, BCL-2, Bax), neuroinflammation (WB, IL-1β, IL-6, TNF-α) and blood-brain barrier integrity (WB,MMP9)** |  |  |
| Sham  SAH  SAH+Didymin  SAH+Vehicle | 0% (0/5)  30% (3/10)  22.22% (2/9)  33.33% (3/9) | 0  2  2  1 |
| **Evaluation of brain water content** |  |  |
| Sham  SAH  SAH+Didymin  SAH+Vehicle | 0% (0/6)  20% (2/10)  12.5% (1/8)  22.22% (2/9) | 0  2  1  1 |
| **Evaluation of neuronal damage (Nissl staining)** |  |  |
| Sham  SAH  SAH+Didymin  SAH+Vehicle | 0% (0/5)  25% (2/8)  12.5% (1/8)  22.22% (2/9) | 0  1  2  2 |
